# Metabolomics and Its Application to Acute Lung Diseases

**DOI:** 10.3389/fimmu.2016.00044

**Published:** 2016-02-29

**Authors:** Kathleen A. Stringer, Ryan T. McKay, Alla Karnovsky, Bernadette Quémerais, Paige Lacy

**Affiliations:** ^1^Department of Clinical Pharmacy, College of Pharmacy, University of Michigan, Ann Arbor, MI, USA; ^2^Department of Chemistry, University of Alberta, Edmonton, AB, Canada; ^3^Department of Computational Medicine and Bioinformatics, School of Medicine, University of Michigan, Ann Arbor, MI, USA; ^4^Department of Medicine, University of Alberta, Edmonton, AB, Canada

**Keywords:** metabolites, nuclear magnetic resonance, mass spectroscopy, pneumonia, acute respiratory distress syndrome, environmental exposure, precision medicine, biomarkers

## Abstract

Metabolomics is a rapidly expanding field of systems biology that is gaining significant attention in many areas of biomedical research. Also known as metabonomics, it comprises the analysis of all small molecules or metabolites that are present within an organism or a specific compartment of the body. Metabolite detection and quantification provide a valuable addition to genomics and proteomics and give unique insights into metabolic changes that occur in tangent to alterations in gene and protein activity that are associated with disease. As a novel approach to understanding disease, metabolomics provides a “snapshot” in time of all metabolites present in a biological sample such as whole blood, plasma, serum, urine, and many other specimens that may be obtained from either patients or experimental models. In this article, we review the burgeoning field of metabolomics in its application to acute lung diseases, specifically pneumonia and acute respiratory disease syndrome (ARDS). We also discuss the potential applications of metabolomics for monitoring exposure to aerosolized environmental toxins. Recent reports have suggested that metabolomics analysis using nuclear magnetic resonance (NMR) and mass spectrometry (MS) approaches may provide clinicians with the opportunity to identify new biomarkers that may predict progression to more severe disease, such as sepsis, which kills many patients each year. In addition, metabolomics may provide more detailed phenotyping of patient heterogeneity, which is needed to achieve the goal of precision medicine. However, although several experimental and clinical metabolomics studies have been conducted assessing the application of the science to acute lung diseases, only incremental progress has been made. Specifically, little is known about the metabolic phenotypes of these illnesses. These data are needed to substantiate metabolomics biomarker credentials so that clinicians can employ them for clinical decision-making and investigators can use them to design clinical trials.

## What is Metabolomics?

**Figure 1:**
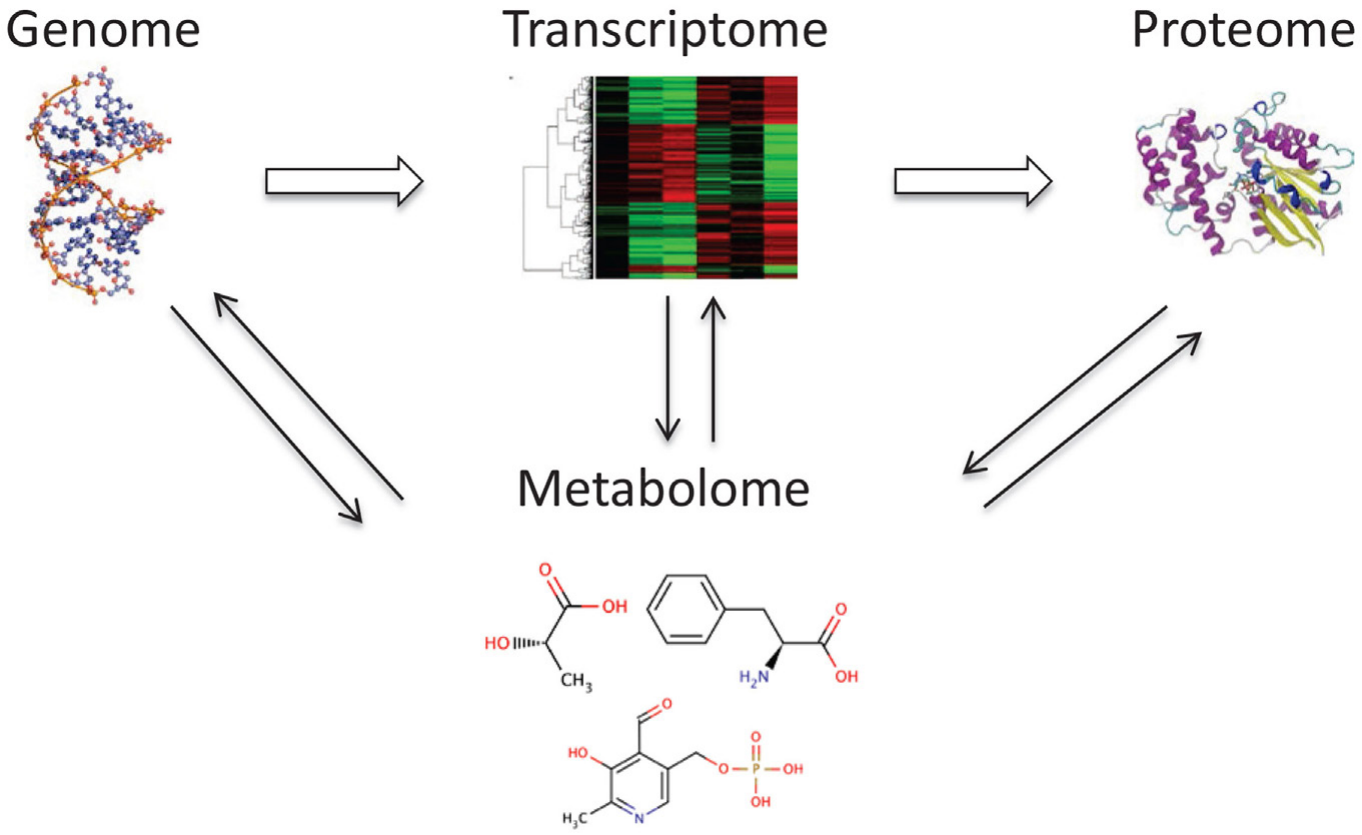
**The metabolome is tightly connected with other “omes.”** The metabolome interacts and reflects the activity of the genome, transcriptome, and proteome.

Metabolomics is a new, rapidly expanding field of systems biology that has garnered significant interest in biomedical research. Its novel aspect involves the ability to generate a “snapshot” measurement of all small molecules, chemicals, and metabolites that may be found in a given sample (1, 2). Because of the ability to analyze small molecules (3), which are a distinct class of compounds from RNA, DNA, and proteins, metabolomics provides a viable alternative to and can complement transcriptomics, genomics, and proteomics. Metabolomics has immense potential for the discovery of novel biomarkers through analysis of continually changing metabolic profiles in response to environmental exposure to toxic substances as well as the manifestation of diseases (4, 5). Metabolomics, also known as metabonomics, can provide a readout of metabolic states in health and disease and identify markers of drug response (pharmacometabolomics). This information is critical for connecting and integrating systems biology sciences (Figure 1).

A key concept in metabolomics is that changes that occur in the transcriptome, genome, or proteome are reflected in the metabolome. These result in alterations in metabolite concentrations in biological fluids and tissues. Interestingly, measurement of metabolites in samples from the human body is not a new notion as metabolic changes have been used as markers since ancient times in the diagnosis of several diseases (6). The diagnosis of diabetes mellitus was based on the sweet taste of urine from patients with Type I diabetes, caused by excessive urinary excretion of glucose as a small metabolite. This led to the development of analytical tools that were implemented more than 100 years ago, and are still in use today, to measure small molecule metabolites in a variety of body samples.

There are several major advantages to metabolomics over traditional clinical chemistry. The first is that advancements in computational technologies allow for the interpretation of metabolite data in the context of its relationship to metabolic pathways (6–8). In addition, recent improvements in the sensitivity and specificity of small molecule detection allow for the characterization and quantification of complex metabolic profiles in biological samples, which result in the simultaneous measurement of dozens, or even hundreds, of metabolites in a single sample (9, 10).

To understand the contribution that metabolomics may make to other fields in systems biology, it is useful to compare the impact that physiological and environmental influences have on genomics, proteomics, and metabolomics. While genomic analysis has identified a number of genes that have effects on the health status of the human body, proteomics has found comparatively fewer proteins, and still fewer disease-associated metabolites have been validated for clinical applications using metabolomics. However, because the metabolome is much more dynamic than either the genome or proteome, metabolomics has the ability to detect changes in metabolites resulting from physiological and/or environmental events over shorter time scales (11, 12). This makes metabolomics a powerful approach for the detection of temporal physiological changes in real time and allows its use as a monitoring approach for potential environmental insults, disease progression, or drug response. In this way, for example, it is possible to monitor time dependent, infection-induced changes in metabolites due to various strains of pneumonia-causing bacteria, which return to levels associated with health upon resolution of infection (13). This level of detail could be particularly important for driving efforts in precision medicine for which reliable and reproducible biomarker credentials (14) are needed for well-informed clinical decision-making and the design of clinical trials (15).

## Biomarkers Arising from Systems Biology Approaches

There are numerous metabolomic and clinical chemistry studies that reproducibly demonstrate that metabolites are highly predictive for a large proportion of complex diseases (16). Thus, metabolomics offers significant opportunities for the advancement of biomarker discovery and analysis in disease diagnostics. Furthermore, exposure to drugs and environmental insults is readily assessed and monitored over time by the application of metabolomic analysis to a wide variety of body samples including saliva, nasal lavage, exhaled breath condensate, sweat, blood, plasma, serum, urine, and feces, among many others (3, 17). Examples of metabolite biomarkers include glucose, used to diagnose diabetes, creatinine to detect kidney disease, cholesterol and triglycerides to determine the risk of cardiovascular disease, uric acid to detect gout, and thyroxine hormone to indicate hypo/hyperthyroidism (6, 16).

We have historically adhered to the concept that each disease can be monitored or diagnosed with a single biomarker. However, this limits the accuracy, precision, and sensitivity/specificity of the detection and diagnosis of disease or changes in the environment. New and developing systems biology technologies and the wealth of information acquired about any given patient (18) suggest that we may be able to use a compilation of biomarkers to describe a given disease, which then greatly enhances disease detection and environmental changes. Here, we discuss the potential applications for how to perform metabolomics analysis. In addition, we summarize the current understanding of metabolomics analysis of community-acquired pneumonia and acute respiratory distress syndrome (ARDS). We also explore the potential for metabolomics analysis of biological samples from healthy individuals exposed to environmental toxins that may result in acute respiratory diseases.

## Metabolomics Study Design, Sample Collection, and Management

### Study Design

As with any scientific study, the design of a metabolomics experiment depends on the scientific question under consideration. A targeted metabolomics approach, where specific metabolites are measured, is best suited for testing specific hypotheses, whereas untargeted approaches that measure all detectable compounds are most often used for hypothesis-generating studies.

The choice of model system (e.g., human, animal model, mammalian cell culture) determined by the experimental question also has implications for study design and sample size. For example, inter-individual variation in most animal studies, where the genetic background, diet, and other environmental factors are relatively homogeneous and can be easily controlled, is minimized. Since these factors cannot be easily controlled in clinical cohorts, human studies usually require larger sample sizes. Clinical variables have to be carefully matched between cases and controls. These include age, weight/body mass index, sex, diet, medication, smoking history, etc., which have been discussed in detail in a number of metabolomics review papers (19–23) and most certainly apply to the design of metabolomics studies of patients with acute lung disease. Mammalian cell culture studies, where the sources of variation can be controlled, require a smaller sample size but also have unique considerations (24). These include the decision whether to analyze either cell metabolites (endometabolome) or cell culture media metabolites (exometabolome) or both. Importantly, regardless of the model system used, most metabolomics assays simultaneously measure hundreds or even thousands of metabolites. This makes multiple statistical tests necessary for the analysis of these data (see Statistical Analysis), which can lead to high false discovery rates (FDR) (25). Various statistical approaches can be used to account for the errors introduced by multiple hypothesis testing, which also makes the number of detected metabolites an important factor in determining the appropriate sample size for a metabolomics study.

For the understanding of new diagnostic and prognostic approaches in metabolomics analysis of acute lung diseases, it is important to consider design options for cross-sectional and other types of clinical studies (23). Patient selection must include a matching of cases/controls that consider confounding factors, for example, factors that influence both the disease state and biomarker concentrations. In addition, a sample size calculation should be carried out with sufficient numbers for internal and external validation to avoid false discoveries in metabolomics (25).

#### Sample Collection, Handling, and Storage

The most critical aspect of sample collection is consistency. This becomes particularly important for the studies that span considerable periods of time like clinical trials that can be conducted over several years. A standard operating procedure for sample acquisition, processing, and storage should be developed prior to study implementation and followed judiciously by all study personnel. The most common problem is variation in the duration of time that a sample sits at room temperature before it is stored (26). Following collection, samples should be kept cold or frozen and stored (preferably −80°C) as soon as possible to minimize metabolite degradation. Sample stability varies widely between different sample types (27–32). In addition to expeditious sample handling, general sample handling practices (e.g., avoiding unnecessary freeze/thaw cycles) should be followed (33, 34). Other considerations for animal studies include variation introduced by anesthesia or euthanasia at the time of sample acquisition. For example, Overmyer et al. showed that use of continuous isoflurane in mouse models led to more consistent metabolomics data compared to other methods of anesthesia or euthanasia (35).

Most biological samples, with the exception of urine (17, 36), require the removal of macromolecules by either chemical extraction (e.g., methanol) or filtration in advance of metabolomics assay (9, 21). Over the past several years, specific protocols have been developed for processing different types of biological samples (30, 37–39). We refer the readers to these references for specific details on these protocols.

Pooled quality control/quality assurance (QC/QA) samples must be included in the sample train to gage variance in data acquisition. These QC/QA samples should be measured as one in every 10 samples of the sample order, and their peak heights and positions compared between measurements to ensure that the quality of data is robust throughout sampling. Ideally, two sets of QC/QA sample should be obtained, with one set containing signals that approximate a negative control (e.g., a control group with baseline signals), and the second set containing signals that resemble a positive control (e.g., a test group with maximally differing signals because of changed conditions) (25, 40).

## Analytical Techniques

Metabolites can be measured by a number of different techniques but the primary analytical platforms that are used in metabolomics are mass spectroscopy (MS) and one dimensional (1D) proton (H) nuclear magnetic resonance (NMR) (21, 41, 42). There are advantages and disadvantages to each and, importantly, no single method captures all classes of metabolites present in the metabolome (9). The type of sample or biofluid can also influence the choice of analytical technique (26, 29, 43). A brief overview of these methods is presented below; more detailed descriptions of these platforms have been recently published (42, 44–48).

### Nuclear Magnetic Resonance

Single proton NMR (^1^H-NMR) involves the use of a large and powerful magnet to align protons that are present in a sample that is placed in an NMR glass tube. There are several types of magnets that can be used, ranging from 400 to 900 MHz. The higher the value, the more sensitive the magnet is to lower concentrations of metabolites or proteins in a sample. Magnets may be equipped with a robot sample handler, which allows users to sequentially assay and to automatically analyze samples without the need to manually insert samples into the magnet at the end of each spectral run.

**Figure 2:**
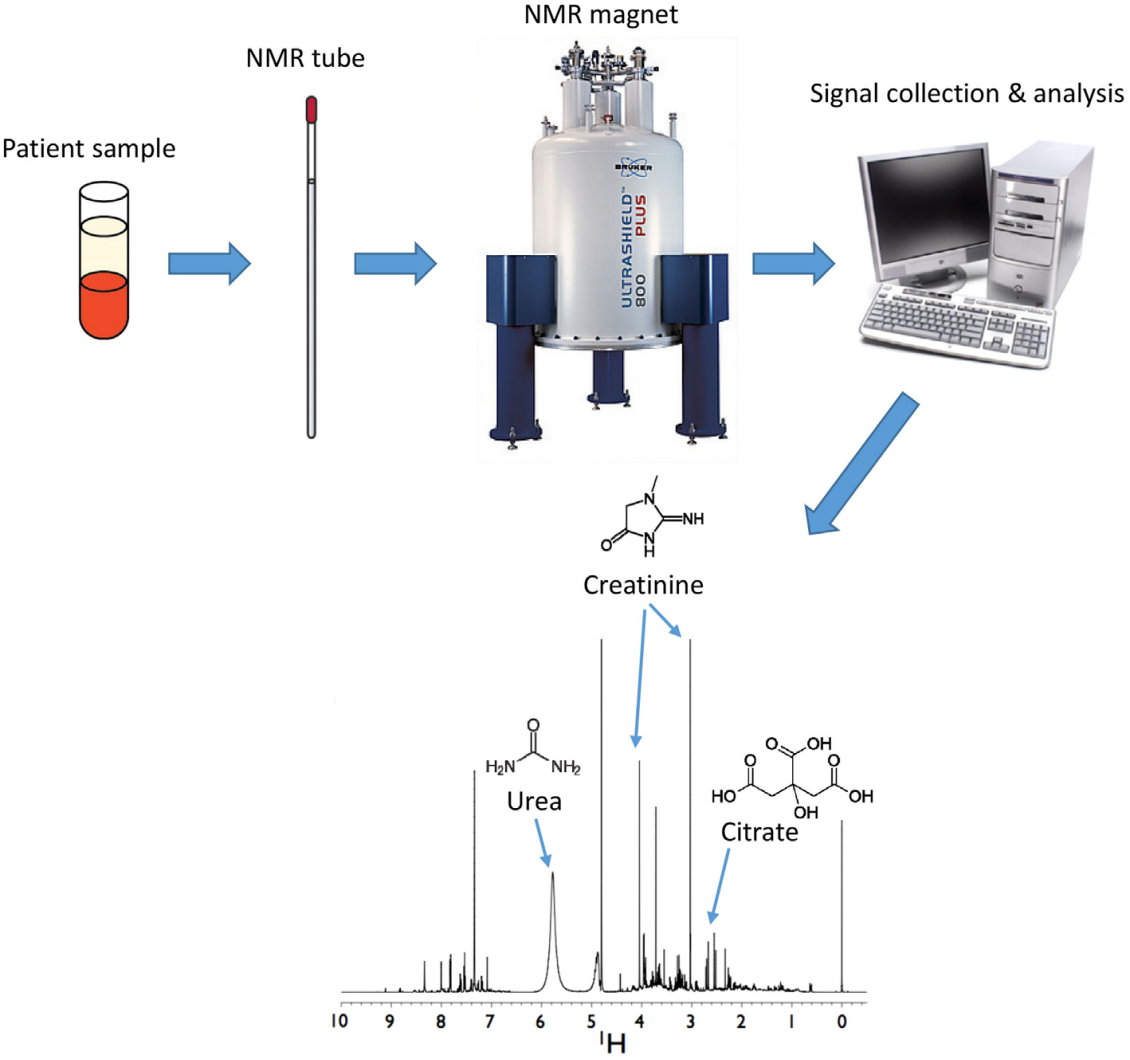
**Analysis of metabolites by nuclear magnetic resonance (NMR)**. Samples are inserted into a magnet from which FID data are collected and analyzed to generate spectra. Positions of metabolites are determined by multiple peaks occurring across a spectrum that correspond to purified standards for each individual metabolite. Areas under the peak curve correspond to the concentration of the metabolite. Shown here is a human urine sample with urea, creatinine, and citrate shown as a few examples of metabolites present in the sample.

Proton NMR is based on the principle that protons resonate in a high magnetic field. A high power short duration radio frequency pulse causes the absorption and subsequent release of electromagnetic radiation, which varies for a compound based on the location (e.g., energy state) of its associated protons. This leads to the generation of a small NMR response, also known as a free induction decay (FID). When the FID is Fourier transformed (49), these signals are translated into peaks that are displayed across a spectrum with units of parts per million (ppm) to distinguish their positions (i.e., chemical shift) (Figure 2). The chemical shifts of these peaks are affected by the proximity of electronegative groups such as nitrogen, oxygen, carbonyls, double bonds, halogens, etc., which influences the place of each type of proton on the spectrum. Every metabolite has its own unique NMR spectrum that represents the environment of each proton. These resonances are further split by interaction with protons on neighboring carbon atoms. The area under the peak is directly proportional to the concentration of each metabolite, which can be calculated with the use of an appropriate internal standard (e.g., DSS).

Consistency in the NMR pulse sequence is a key. As long as the same methodology (i.e., field effect pulses, gradients, delays, power levels) is used, and the method components are properly calibrated for delivered performance, then the result should be evaluated on solvent suppression and any residual or unexpected stray suppression throughout the rest of the spectrum. The optimal NMR pulse sequence is the one that works consistently for the respective instrument and is one that can be reliably reproduced. In addition, the type of spectral analysis software that will be used, such as Chenomx software,^1^ for determining the identities and concentrations of metabolites in a spectrum may also influence the choice of pulse sequences.

The advantage of using NMR is that almost every biological compound has a distinct and reproducible NMR signature. This makes it possible to calibrate the magnet for each compound using purified standards. Each compound gives either single or multiple peaks, depending on the number of protons present in the molecule if using ^1^H-NMR. Metabolite detection by NMR is unique in that it is non-destructive to the sample, and in some cases, it is possible to return samples (e.g., urine) unaltered to the investigator following assay. This allows conformation by other techniques or re-testing later if desired.

It can be deceptively difficult to have multiple instruments, possibly in quite distant facilities, provide accurate and precise results for comparisons, but it can be done (36). If the instrumentation is well understood and operated by a knowledgeable spectroscopist, then after the initial investment of setup time, consistent data should be relatively easy to obtain. The primary spectroscopic requirements are that the pulse sequence components (e.g., excitation pulse, power levels, and tune/match) are properly calibrated for delivered effect at the probe head. Proper use of controls at regular intervals then will lend confidence in the long-term performance.

### Mass Spectroscopy

**Figure 3:**
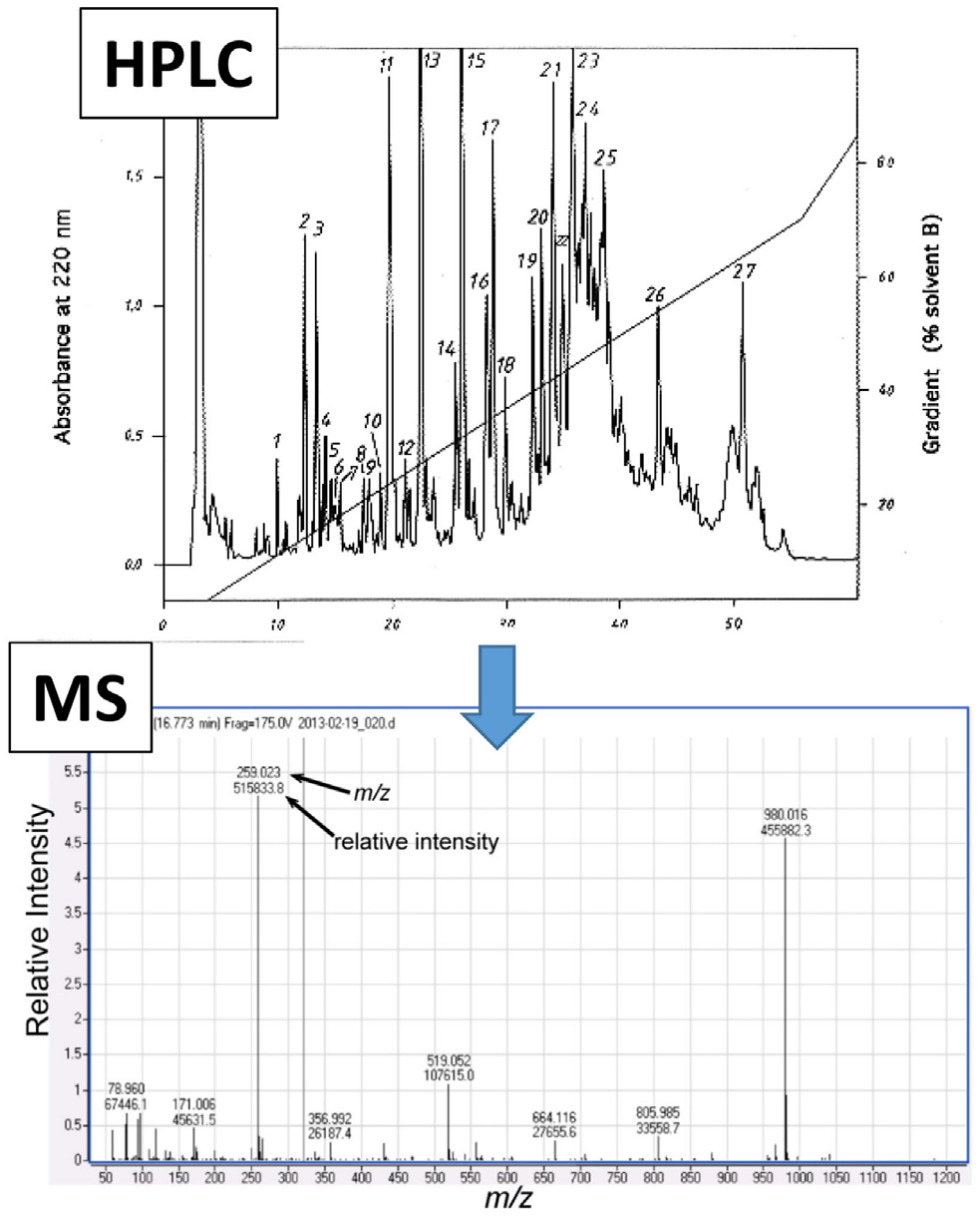
**Representative mass spectroscopy (MS) spectrum following high performance liquid chromatography (HPLC)**. Initial data that are generated from liquid chromatography (e.g., HPLC, shown as an example in upper panel) which is often conducted prior to MS analysis (lower panel). The MS spectrum shows numerical values that correspond to the mass-to-charge ratio (*m/z*, *x*-axis) and relative intensity (*y*-axis) for each detected metabolite.

**Figure 4:**
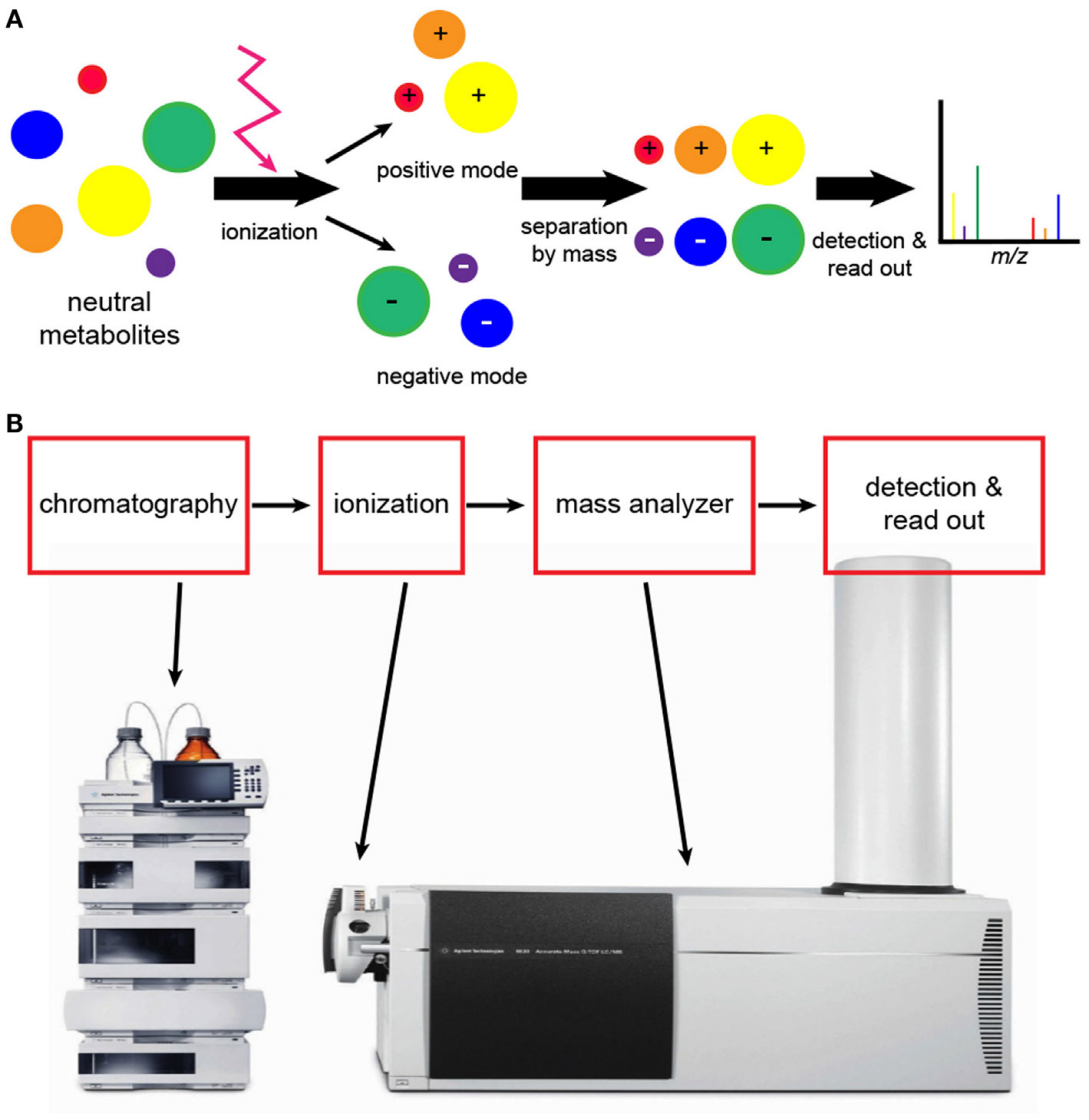
**Analytical workflow of liquid chromatography (LC)-mass spectroscopy (MS)**. **(A)** An illustration of what happens to molecules during LC-MS. Neutral molecules may be ionized using a number of different techniques, but electrospray ionization is frequently used. Following ionization, negatively and positively charged compounds are generated. LC-MS conducted in negative and positive modes will detect negatively and positively charged ions, respectively. The read-out is a graphic representation of compounds as shown in Figure 3. **(B)** Elaborate equipment is needed to conduct LC-MS metabolomics. The initial step is chromatography followed by ionization and mass analysis of the molecules.

Mass spectroscopy generates metabolite spectral data as mass-to-charge (*m/z*) ratios and relative intensities (41), but quantified data can be generated with the use of compound standards (Figure 3). For metabolomics studies, MS is most often preceded by either liquid chromatography (LC) or gas chromatography (GC) (Figure 4).

#### Liquid Chromatography-Mass Spectroscopy

Liquid chromatography-MS is the analytical approach that is most often used for metabolomics studies because it allows the detection of a broad range of different classes of metabolites (33, 45, 50). There are a number of advantages to the use of LC-MS for metabolomics. It is sensitive to nanomolar concentrations; there is no need for sample derivatization (see GC-MS), and there is good coverage of mass range, which permits the detection of metabolites with different chemical properties. In addition, aqueous and lipid metabolites can be simultaneously assayed, and advancing technology is permitting greater separation and detection of metabolites including lipids (42). The disadvantages of LC-MS include its high variability, particularly across instruments that it is not routinely quantitative, and there is no standardized metabolite library (21).

A critical component of the LC-MS assay is the type of chromatography column that is used because it determines the types of metabolites that will be detected (42, 51). In addition, the polarity and pH of the solvent that is used to move the sample through the LC column influences sample retention. Reverse-phase columns, like C18 columns, provide good retention and separation of non-polar compounds (33, 42, 45, 51). Alternatively, hydrophilic interaction chromatography (HILIC) columns are better for the detection of polar compounds. Recent advances in LC-MS include the introduction of ultra performance liquid chromatography (uHPLC) (52), which detects smaller sized particles and has led to better peak capacity, greater resolution, and higher throughput due to shorter sample run times and capillary electrophoresis (CE)-MS (48, 53), which has the capacity to separate complex mixtures with high resolution and minimum sample manipulation.

For the detection of metabolites by LC-MS, the sample must be ionized. The mass analyzer then determines the mass of the ionized compounds, which is reported as the *m/z* ratio (Figure 4). There are a number of different techniques for ionization, but electrospray ionization (ESI) is widely used because it generates both positive and negative ions (41, 45). Atmospheric pressure chemical ionization (APCI) is slightly less sensitive but works well with non-polar compounds such as lipids. For complex samples, matrix-assisted laser desorption/ionization (MALDI) is very useful and is highly sensitive, and it is the preferred approach for higher mass compounds. The primary disadvantage of MALDI is background interference, particularly with lower molecular weight compounds.

There are a number of options for the types of mass analyzers for coupling with LC (42, 46). The most common mass analyzers are the quadrupole, time of flight (TOF), and ion trap analyzers. Due to their relatively low cost, quadrupole analyzers are widely used. Triple quadrupole (QQQ) analyzers, in which three quadropoles are combined in succession, allow for MS/MS, or further fragmentation of ions during analysis. TOF analyzers determine the *m/z* by accelerating ions and then measuring the time it takes to travel down a flight tube. TOF analyzers have high mass accuracy, are highly sensitive, and quickly acquire data. They can be coupled with a quadrupole (Q-TOF), which is well suited for metabolite detection. Ion trap analyzers are similar to quadrupoles because they allow for detection of particular ions and are affordable. They trap ions of interest and accumulate them for better sensitivity or they can trap and fragment a specific ion multiple times; this is referred to as MS^n^ but ion trap analyzers do not have the broad capabilities of QQQ analyzers. Newer technologies like Fourier transform ion cyclotron resonance (FT-ICR) have the highest degree of mass accuracy and have MS/MS and MS^n^ capabilities but are limited by high cost.

#### Gas Chromatography-Mass Spectrometry

**Figure 5:**
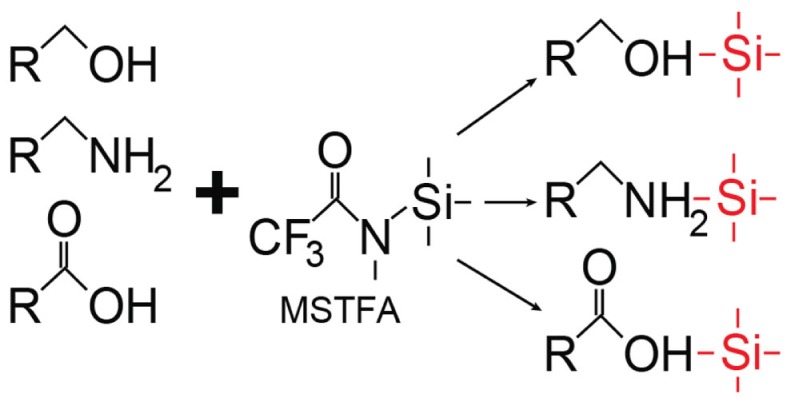
**Representative scheme of silylation**. In this case, silylation using *N*-methyl-*N*(trimethylsilyl) trifluoroacetamide (MSTFA) as a type of derivatization can be done in preparation for gas chromatography (GC)-mass spectroscopy (MS).

The advantage of GC-MS is that it is highly sensitive and specific for separation and detection of volatile metabolites such as organic acids (42, 44). In addition, spectral patterns and retention times of compounds are highly reproducible, which allows for the use of established compound libraries. Also, there is lower instrument-to-instrument variability, which is a limitation of LC-MS. However, the use of GC-MS for metabolomics study is reserved for thermally stable volatile compounds that are of low polarity and primarily those that are amenable to derivatization, which aids in making compounds less polar and more stable. This process can lead to loss of metabolites, and incomplete derivatization can add spectral artifacts. Most often, chemical derivatization is performed using oximation reagents such as hydroxylamines or alkoxyamines, which react with aldehyde and keto groups. This is followed by silylation with *N*-methyl-*N*(trimethylsilyl)-trifluroacetamide (48); it can also be achieved with silylation alone. Silylation involves the replacement of hydrogens in functional groups (e.g., –COOH) with a trimethylsilyl group [−Si(CH_3_)_3_] (54) (Figure 5). For GC-MS metabolomics studies of organ tissue, *N*,*O*-bistrifluroacetamide with trimethylchlorosilane has been used (48).

In GC-MS, a carrier gas propels the sample through the separation column, after which it is ionized by electron ionization (EI) or chemical ionization (CI) for detection by the mass spectrometer. EI is the most frequently used ionization technique, and mass analyzers are those which were described for LC-MS.

#### Applications of Capillary Electrophoresis for Metabolomics

Capillary electrophoresis, although used less frequently, presents a viable option for the detection of metabolic markers. It separates complex mixtures with high resolution and minimum sample treatment. A wide range of polar metabolites and ionic compounds are amenable to CE separation, which makes it a complementary tool to the LC and GC techniques described above. CE is often used in combination with EI-TOF-MS. Combining CE with MS is rather challenging, which limits the applications of this separation method (48, 55). Nevertheless, CE has been successfully applied for the identification of metabolic markers in serum, urine, cerebrospinal fluid, and cell lines (56–58). Naz et al. recently published a CE-TOF-MS method that allowed identification of metabolic markers in an experimental model of ventilator induced lung injury (VILI) (53). Thus, MS coupled with chromatography represents a diversity of applications that may be useful for the detection and differentiation of diseases and environmental impact on clinical and experimental biofluids.

## Analysis of Metabolomics Data

Analysis of metabolomics data encompasses a number of operations from initial processing used to perform quality assurance and quality control, imputation of missing data, normalization, and statistical analysis, to biological data interpretation. Initial data processing is platform specific and varies widely for the analytical platforms described in previous sections. Most instrument vendors provide proprietary software for processing raw data that often include options for data normalization and basic statistical analysis. LC-MS, GC-MS, and NMR data processing have been extensively reviewed (6, 12, 42). Significant progress has been made in recent years to increase accuracy and reproducibility of LC-MS and GC-MS data and to automate processing of NMR data; however, there are still many unresolved issues. In general, the analysis of targeted metabolomics data is usually more straightforward. Analysis of untargeted metabolomics data, where not all metabolites are identified, is much more complex. In this section, we will primarily focus on the methods and tools for performing statistical analysis, biological data interpretation, and the identification of potential biomarker candidates.

### Statistical Analysis

Statistical analysis is a critical part of any high-throughput study, and metabolomics is no exception. Several common types of analyses involve finding metabolites/features that differentiate experimental and control groups, and determining the extent of associations between metabolites and phenotypic or clinical variables. An important concept that became particularly apparent from gene expression profiling studies is the necessity to validate findings using a separate group of samples obtained from a different independent population (25). This becomes particularly important for building various classificatory and predictive models, which are the required step in biomarker discovery and the validation of biomarker credentials (14, 59). The choice of analytical technique has implications for the number of samples that should be collected, including biological and technical replicates, the type of controls, and other factors that may influence study outcome. As mentioned above, another important factor to be considered when choosing the appropriate sample size is that the biological variability of the metabolome is higher in the human population compared to well-controlled animal studies. All these parameters should be considered at the early stages of experimental design.

One common feature of all analytical techniques described above is that they produce complex multi-dimensional data sets. Therefore dimension reduction techniques, such as principal components analysis (PCA) and various clustering methods (e.g., hierarchical, or k-means clustering), provide a useful tool for the initial survey of the global properties of the data. For example, PCA is an approach that is frequently used to identify potential outliers and assess the overall quality of the data. Parametric statistical tests, such as the Student’s *t*-test for two experimental groups, or ANOVA for multiple groups, are often used to identify differentiating metabolites.

Given the large number of metabolites that can be measured in a single experiment, multiple tests have to be performed, increasing the probability of type I error (false positives). To remedy this problem, test results have to be adjusted using family wise error rate or FDR (60, 61). In addition to these tests, fold change analysis is frequently used to determine the magnitude and direction of the change.

**Figure 6:**
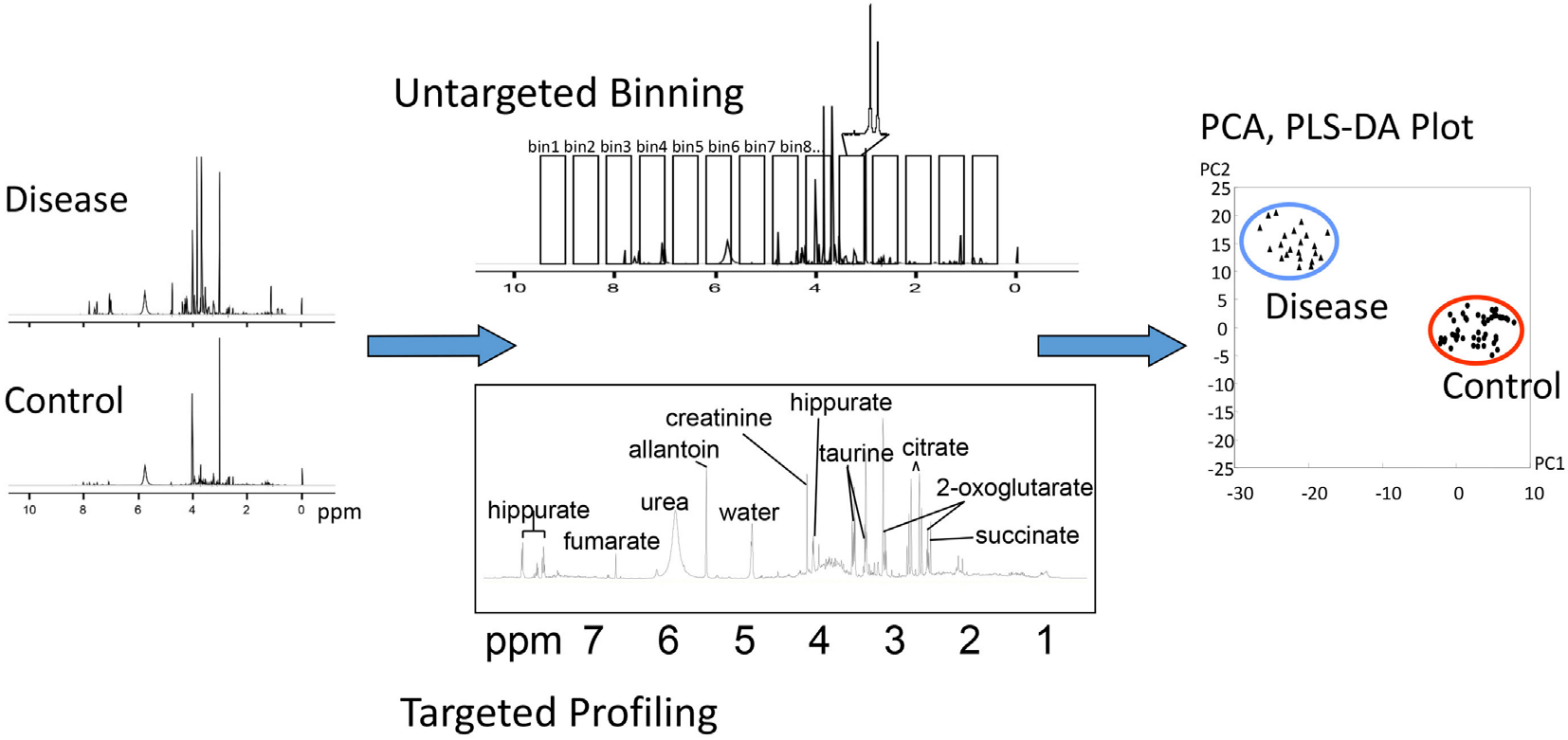
**Different methods of analysis of metabolomic data**. In this example, NMR spectra collected from control and diseased subjects may be analyzed by untargeted “binning” or targeted profiling, either of which can be subjected to PCA or PLS plotting.

It is worth mentioning that statistical analysis can be performed on either absolute concentrations or relative peak intensities and does not require prior identification of metabolites, which is the basis of the chemometric, or “untargeted,” approach (62, 63). Despite recent progress in data processing algorithms, identifying and quantifying all peaks in a given NMR spectra or all features from a GC-MS or LC-MS experiment remain a time-consuming and challenging task. A chemometric approach provides a viable alternative. For example, NMR spectra can be divided into “bins” of equal chemical shift intervals, often referred to as “binning” (Figure 6). The area of each bin is integrated, and statistical analysis can be performed to identify the spectral regions that differ between groups. These results can then be used to identify specific metabolites that contribute to the signal in that region. Untargeted GC-MS studies, and especially LC-MS studies, are characterized by the presence of multiple unknown features, some of which may be strongly associated with the disease or specific biological condition under study. Statistical analysis can be performed on those followed by computational and experimental analysis to verify their identity.

Chemometric approaches have been broadly used in animal and human studies for identification of disease biomarkers (19, 64), as well as for assessing drug metabolism and drug safety (65). The advantage of chemometric methods is that they provide a practical way to deal with large volumes of data. An alternative approach, where quantitation and identification of the broad range of metabolites is performed up-front, also has merits; it permits the advantageous use of parametric statistics, pathway analysis, and hypothesis-generating tools that are described below and has the potential to provide broader context for data analysis.

### Knowledge-Based Methods for Biological Data Interpretation

Irrespective of the specific technique used, the output of statistical analysis is usually a list of metabolites that are significantly associated with a phenotype. A growing number of metabolomics as well as genomics and proteomics studies have shown that gaining biological insight from a list of differentially regulated molecules is challenging (25, 34).

**Figure 7:**
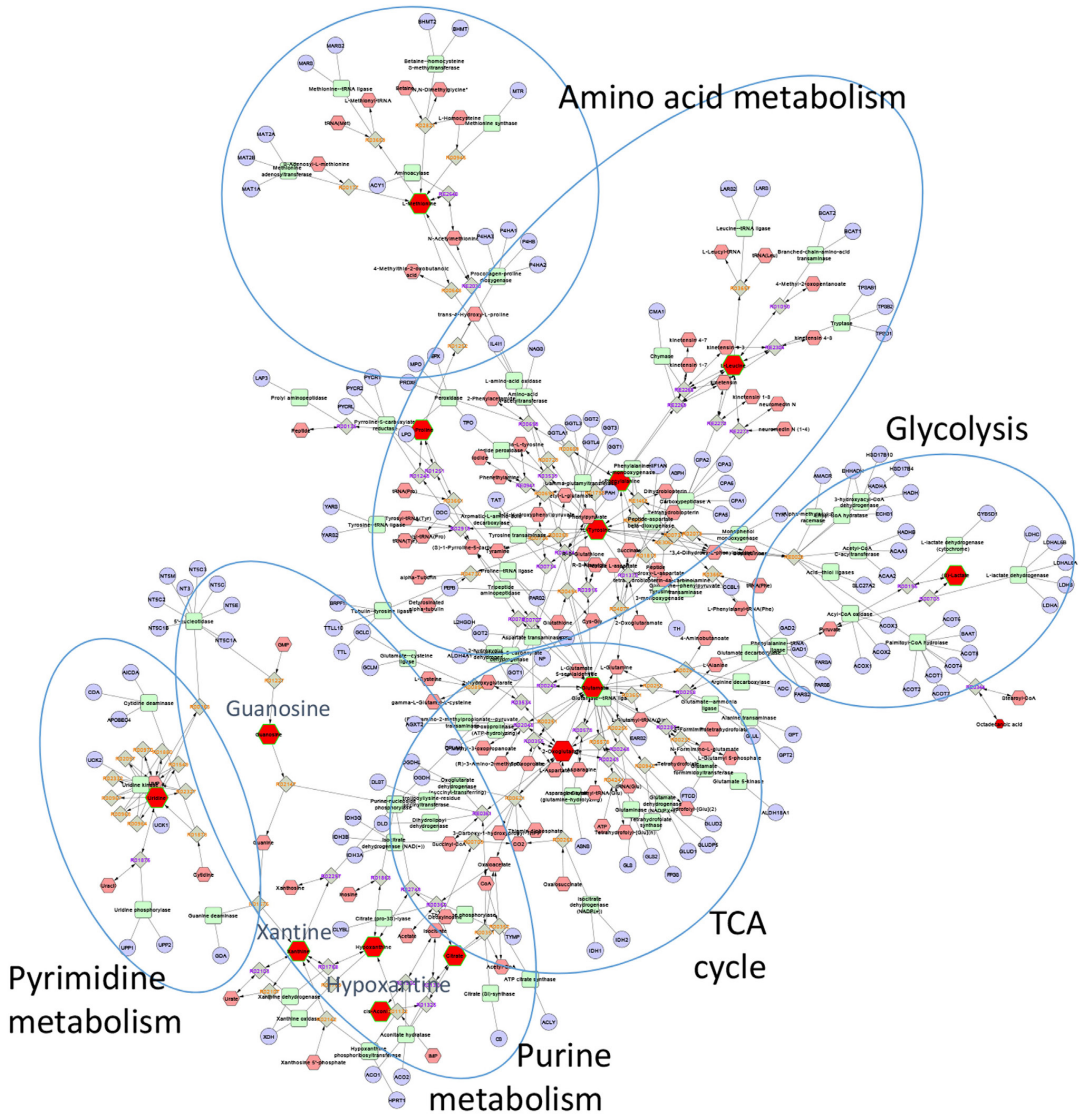
**Metscape network showing metabolites that differentiated ARDS BAL fluid samples from those of healthy controls**. Red nodes represent experimentally measured metabolites that were used by Metscape as seeds for building the metabolic network. The program also provides information about metabolic reactions (gray nodes), metabolic enzymes (green nodes), and genes (light blue nodes). The most significant BAL metabolites of ARDS were those associated with purine metabolism, specifically hypoxanthine, xanthine, and guanosine.

The first step in this process usually involves mapping known metabolites onto biological pathways. A number of well-­documented public databases contain carefully curated information about metabolites, metabolic reactions, enzymes, genes, proteins, and pathways (66–68). A number of open source (69–73), and commercial tools (MetaCore, Ingenuity Pathway Analysis) make use of pathway information and provide various ways to map experimentally observed changes onto metabolic pathways. To illustrate the application of pathway mapping for the analysis of metabolomics data, we recently used published untargeted LC-MS profiles of bronchial lavage fluid from patients with ARDS and healthy controls (Figure 7) (51). This study identified 26 metabolites that were significantly different between the two conditions. We loaded these compounds into the pathway-mapping tool Metscape^2^ (73). Metscape is a plugin for a widely used open source network analysis and visualization tool Cytoscape^3^ (74). It supports network-based exploration of metabolomics and gene expression data. Figure 7 shows a metabolic network for a subset of these compounds. Placing compounds into metabolic pathways helps connect the observed changes to previously reported biological observations. For example, Evans et al. reported a fourfold increase in guanosine and 41- and 19-fold increases in hypoxanthine and xanthine in ARDS (51), respectively and pointed out that these findings can be related to previously reported inflammatory effects of uric acid, which is a product of guanosine metabolism (75, 76).

In addition to mapping metabolites to pathways, it is often useful to be able to assess the relative significance of different pathways. This task can be accomplished through enrichment analysis. The goal of such analysis is to evaluate what pre-defined biologically meaningful sets of metabolites (e.g., pathways) are enriched with differentially regulated metabolites from a given experiment. This approach was originally developed for the analysis of gene expression data (77) and recently applied to metabolomics data (78–80). The output of enrichment analysis is usually a ranked list of pathways or other biological categories (e.g., Gene Ontology terms) and the list of experimental compounds mapped to them. While enrichment testing is a well-established data reduction technique that proved to be invaluable for the analysis of microarrays, *RNA seq* and proteomics data, applying it to metabolomics has some challenges. Metabolite enrichment testing usually has lower statistical power than gene enrichment testing due to the relatively small number of identified metabolites measured in a given study. Metabolomics data are considerably sparser than gene expression data, which also complicates the analysis. The problem can be further compounded by metabolites involved in multiple metabolic pathways (e.g., ATP, NADP, NADPH, etc.).

One important limitation of all techniques that rely on pathway mapping is relatively low coverage of experimentally measured metabolites included in pathway databases. The best represented classes of metabolites include intermediates of primary metabolism, whereas the coverage of secondary metabolites and lipids is significantly lower (81). Lack of standard unique metabolite identifiers creates additional technical challenges for pathway mapping. In recent years, several approaches have been described that attempt to overcome this problem and expand the number of metabolites that are included in secondary analysis. For example, MetaMapp combines the biochemical reactions from KEGG with Tanimoto chemical and National Institute of Standards and Technology (NIST) mass spectral similarity scores to build extended metabolite networks (81). A recently published tool MetaMapR takes this approach one step further and allows users to calculate structural and mass spectral similarity directly within the program and supports interactive network visualization (82). Other efforts to overcome some of these problems involve generating automated annotations by linking compounds to publications via Medical Subject Headings (MeSH) (83).

### Data-Driven Data Analysis Methods

One of the characteristic features of metabolomics data, generated through untargeted LC-MS and GC-MS studies, is the presence of multiple unknown features that are excluded from pathway analysis. Data-driven approaches that allow the inclusion of unknown features into secondary analysis are rooted in an observation that functionally relates metabolites tend to display correlated changes. Early work in this area utilized Pearson’s correlation coefficients to establish linear associations between metabolites (84). However, Pearson’s correlation does not differentiate between direct and indirect associations, and metabolism is not inherently linear. Subsequently, several groups proposed using Gaussian graphical modeling to reconstruct partial correlation networks among sets of genes or metabolites to overcome these limitations (85–87). While these methods have potential to complement knowledge-based data analysis methods described above, practical application may be limited by the number of analyzed samples.

In NMR analysis, presently a semi-automated approach [Chenomx software (see footnote 1)] seems to be the most trusted and reliable form of analysis for NMR spectra, whereas fully automated analysis software is under rapid development [e.g., Ref. (88)]. The Metabolomics Society also lists available software packages.^4^

### Relative Sensitivities of NMR and MS-Based Approaches

**Figure 8:**
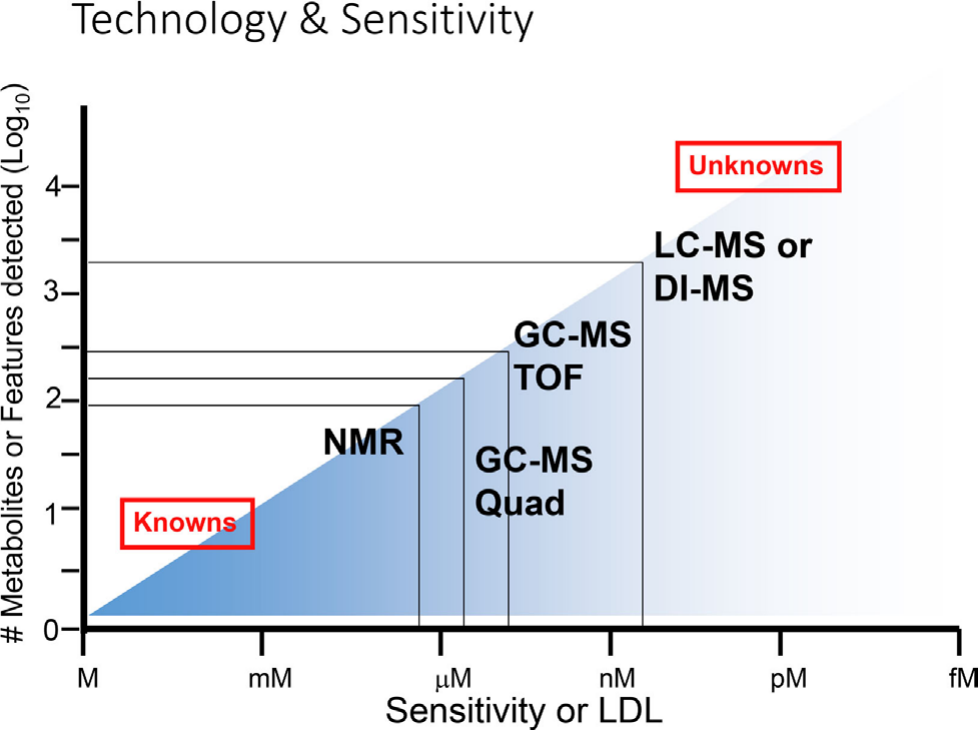
**Range of sensitivities of metabolomic technologies**. At the lower end of sensitivity or lower detection limit (LDL), NMR is suitable for detection of smaller numbers of known metabolites, while at the higher end of sensitivity (at right), MS-based technologies are superior for detection of known as well as unknown metabolites. Adapted with permission from Wishart (6).

The relative sensitivities of NMR versus MS-based approaches are a central issue in the decision-making behind which technology to use in biological studies. While NMR is a preferred approach for the detection of a broad spectrum of metabolites, its ability to detect low concentrations of metabolites is limited compared to MS-based analyses (Figure 8). While NMR is suitable for the majority of known metabolites, the limit of detection of NMR is usually in the millimolar to micromolar range (21). However, recent developments in NMR-based approaches have shown improved sensitivity in the nanomolar to micromolar range. In contrast, MS-based approaches can detect metabolites at less than picomolar levels, which substantially increases the number of metabolites or features detected in a given sample. The problem is that many of these very low abundance metabolites are not well characterized, and they give rise to a large number of “unknown” features. In general, it may be best to initiate a high-throughput analysis, for example when searching for potential biomarkers in biological fluids, using an NMR-based approach in a pilot experiment. Once a limited number of marker metabolites are identified, these could be pursued further using more sensitive MS-based approaches.

## Applications of Metabolomics to Acute Lung Diseases

### Pneumonia in Animal Models and Humans

*Streptococcus pneumoniae* is a major cause of bacterial infection in the lower respiratory tract and is the most common cause of community-acquired pneumonia (89). Millions of people in North America are affected by pneumonia, and this illness results in over half a million hospitalizations each year (90). The accurate diagnosis and antibiotic treatment of this disease at the individual level are of primary importance in controlling the incidence of pneumonia. Systems biology approaches are hoped to improve diagnosis and facilitate monitoring of disease together with prescribing appropriate therapy in pneumonia and similar inflammatory lung diseases (91).

**Figure 9:**
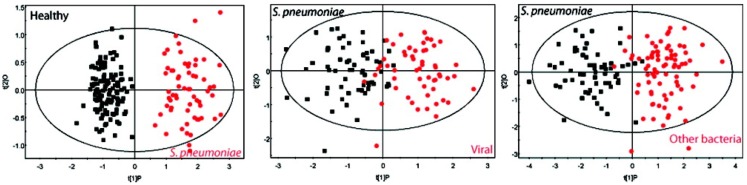
**Differentiating between different types of pneumonia in human patients**. Urinary metabolites were found to be distinct in pneumonia caused by *S. pneumoniae* and other pathogens. These graphs show OPLS-DA models based on 61 measured metabolites found in the urine from *S. pneumoniae* patients compared with those found in viral pneumonia and other bacteria (including *Mycoplasma tuberculosis*, *Legionella pneumophila*, *S. aureus*, and others). Note that the labeling for *S. pneumoniae* is shown in red at left while this is black in the middle and right panels. Adapted with permission from Slupsky et al. (92).

The application of ^1^H-NMR-based metabolomic analysis of pneumonia patient urine samples demonstrated that definitive metabolic profiles specific to *S. pneumoniae* infection could be identified (Figure 9) (13). Notably, the pattern of urinary metabolites in pneumococcal pneumonia was distinct from those associated with pneumonia caused by viruses and other bacterial strains, as determined by orthogonal projections to latent structures (OPLS)-discriminant analysis (DA). In addition, serial collection of urine samples from patients with pneumonia over time demonstrated that infected individuals reverted to a normal metabotype upon resolution of infection, indicating that the specific metabolic profiles in urine were unique to the infection. Blinded analysis of the urine samples showed that NMR-based metabolomic profiling provided excellent sensitivity and specificity, with a high accuracy rate (91%), for identification of *S. pneumoniae* infection. Interestingly, none of the subjects in the blinded sample population were false positives, which would have been predicted with up to 10% colonization of the healthy adult population by pneumococcal strains.

**Figure 10:**
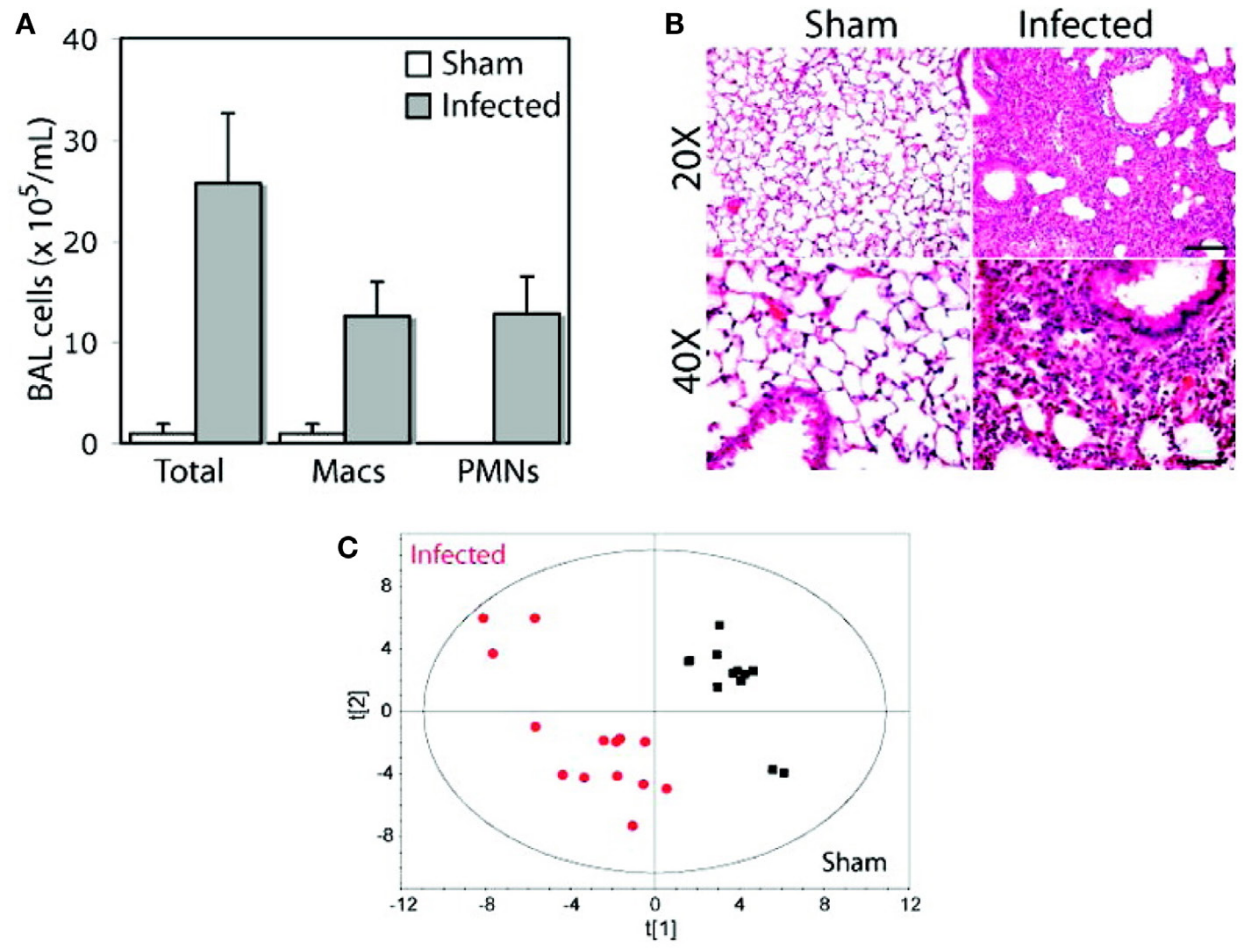
**Distinct metabolic profiles in animals infected with *S. pneumoniae* and *S. aureus***. An inbred strain of mice (C57BL/6), maintained in specific virus antigen-free housing with autoclaved bedding and dietary supplies, was infected intratracheally with a clinical isolate of *S. pneumoniae*, serotype 14. After 24 h of infection, bronchoalveolar lavage (BAL) samples were analyzed for cell counts **(A)** and histology was carried out on lung sections **(B)** to confirm inflammation arising from infection. At the same time, urine samples were collected from animals that were subjected to NMR analysis, and a PCA model of urinary metabolite concentrations was generated **(C)**. Macs, macrophages; PMNs, polymorphonuclear neutrophils. Adapted with permission from Slupsky (13). Copyright 2009 American Chemical Society.

Using an animal model of pneumonia, it was found that distinct urinary metabolic profiles resulted from infection by two different pathogens, *S. pneumoniae* and methicillin-resistant *Staphylococcus aureus*, a major cause of antibiotic-resistant pneumonia that is normally associated with hospital-acquired pneumonia but that has been increasing in the community (92). Following 24 h of infection with *S. pneumoniae* or *S. aureus*, in-bred C57BL/6 mice exhibited significant urinary metabolic changes that could be detected using NMR-based measurements (Figure 10). Urinary metabolic profiles reverted to normal, healthy values upon resolution of infection, suggesting that these metabolites were specific to bacterial infections. These results underscore the potential that metabolomics has for the diagnosis, and monitoring of the antibiotic therapy of pneumonia, and how this could be applied to the clinical management of pneumococcal disease both in community- and hospital-acquired illnesses.

Two recent studies have supported the concept of applying metabolomics analysis to the diagnosis of pneumonia. Both of these applied mass spectrometry (MS)-based approaches and found that a number of urinary and blood metabolites correlated with the incidence of pneumonia infection. In the first study carried out in The Gambia, West Africa, it was found that metabolomic analysis of urine and plasma samples distinguished severe pneumonia patients from community controls in children (93). The specific urinary metabolites found to decrease in children with pneumonia were uric acid and l-histidine while plasma metabolites that were increased included hypoxanthine and glutamic acid. Plasma levels of l-tryptophan and ADP were reduced in children with pneumonia. These six metabolites emerged as markers of key differences between the two groups. The authors speculated that these metabolites are important in the host response through antioxidant, inflammatory, and antimicrobial pathways, as well as energy metabolism. The drawbacks of this study were its small scale (only 11 children with pneumonia were examined), and that metabolite concentrations could not be quantified as the MS-based approach only determined relative changes in metabolites.

In the second study, the global metabolomic profile in plasma from surviving and non-surviving patients (by 90 days) with community-acquired pneumonia was determined (93). This study also used MS-based approaches to identify metabolites in plasma samples and compared these with the presence of inflammatory markers including interleukin (IL)-6, IL-1β, and tumor necrosis factor-α (TNF). A number of metabolites were found that differed significantly between surviving and non-surviving pneumonia patients. In particular, pseudouridine was increased in non-surviving patients, and this was subsequently determined to induce significant TNF and IL-1β production, likely through Toll-like receptor 4 (TLR4), from monocytes/macrophages in culture. These findings showed novel findings regarding metabolite detection in plasma samples in patients that survived pneumonia. These data were acquired using MS-based approaches for which quantitation of metabolites was not done. Nevertheless, taken together, these studies suggest that metabolomics has the potential to diagnosis and track prognosis in patients with pneumonia in the community.

The application of metabolomics should be taken into consideration with clinical decision-making when treating community-acquired pneumonia, which involves determining whether (1) to withhold antibiotics, (2) to use targeted antibiotics, or (3) to stratify patients in order to give more aggressive therapy to those with higher risk (94). Stratification could generate different metabolic markers for pneumonia than diagnostic markers, and this needs to be kept in mind as a potential future study for the metabolomics of pneumonia.

### The Metabolomics of Acute Respiratory Distress Syndrome

#### ARDS Is a Significant Hazard to Human Health

Acute respiratory distress syndrome (ARDS) in adults is characterized by an abrupt infiltration of inflammatory, fibrin-rich exudate into the pulmonary interstitium and airspaces that impairs lung function and gas exchange (95–98). There are a number of conditions that can prompt the development of ARDS but the most common precipitating etiologies include sepsis, pneumonia, and severe trauma.

**Figure 11:**
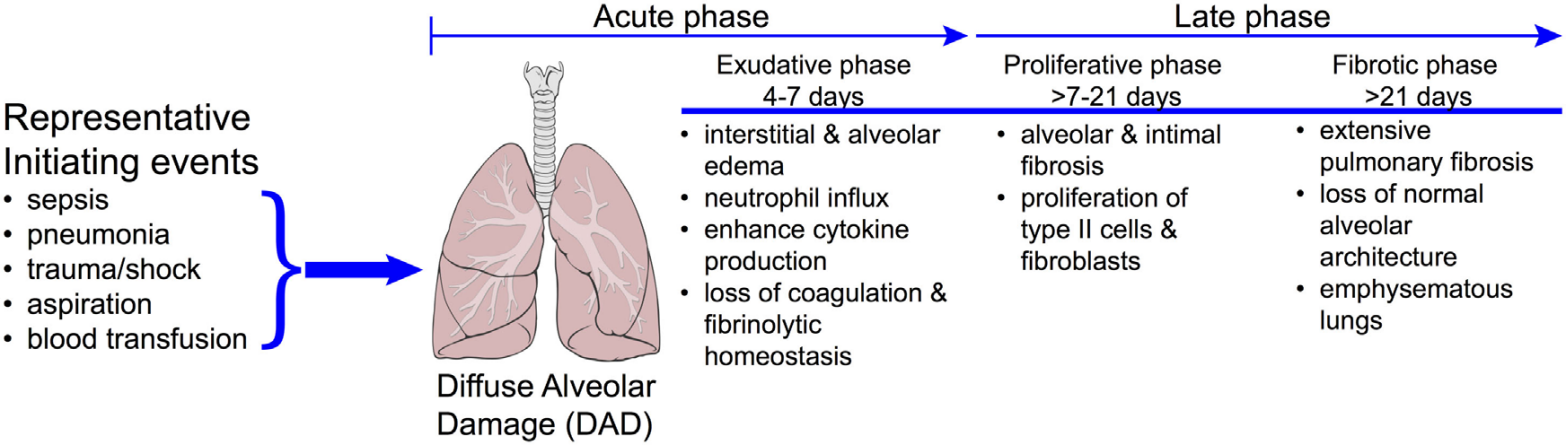
**Progression of disease in ARDS**. The clinically challenging problem of acute respiratory distress syndrome (ARDS) is illustrated by the diversity in the underlying etiologies and the complex time course of the disease. Approximately 40% of patients with severe sepsis will develop ARDS. Patients who do not recover during the proliferative phase may go on to develop emphysematous regions in the lungs and ultimately fibrosis. While it is reasonable to expect that each of these phases will have a distinct metabolomics phenotype, these have yet to be realized. Reproduced with permission from MacLaren and Stringer (104). Illustration of lungs from "Lungs diagram simple" by Patrick J. Lynch, medical illustrator. Licensed under CC BY 2.5 via Wikimedia Commons – http://commons.wikimedia.org/wiki/File:Lungs_diagram_simple.svg#mediaviewer/File:Lungs_diagram_simple.svg.

The early phase of ARDS is characterized by diffuse alveolar damage (Figure 11), an associated increase in endothelial permeability, intravascular thrombi, severe epithelial injury with denudation of alveolar wall basement membranes, and the accumulation of alveolar infiltrates in the airspaces, which are highly enriched with neutrophils (a hallmark of ARDS) (95). In ARDS survivors, these changes progress for several days to a repair phase, which is characterized by hyaline membrane formation, the appearance of mononuclear cell infiltrates, and development of intra-alveolar and interstitial fibrosis (Figure 11) (96). Patients are critically ill, requiring treatment and mechanical ventilation in an intensive care unit setting. As such, the morbidity and mortality associated with ARDS is significant. In the United States, ARDS accounts for an estimated 75,000 deaths per year (99), and overall mortality has been estimated between 20 and 40%. Despite the seriousness of this human hazard, knowledge of the pathogenesis of ARDS is incomplete (100, 101) and to date, there is no effective pharmacotherapy.

Acute respiratory disease syndrome is a clinically challenging problem, due in part to, the disparity in its definition and its heterogeneity. The first consensus definition by the American-European Consensus Conference (AECC) included the sub-category of acute lung injury (ALI), which used the same criteria as ARDS but with less severe hypoxemia (PaO_2_/FIO_2_ of <300 mm Hg). In 2012, the definition was further refined by the European Society of Intensive Care Medicine, which resulted in the generation of the Berlin Definition of ARDS (Table 1); it has been endorsed by the American Thoracic Society and the Society of Critical Care Medicine (102). Notable changes include the removal of the sub-category of ALI and the addition of more detail about levels of oxygenation and mechanical ventilation. Utilization of the Berlin Definition of ARDS is expected to allow for greater delineation of patients with ARDS for inclusion in clinical trials (103), but it does not fully address the heterogeneity of the disease that originates from a broad range of underlying etiologies (104, 105). These problems have undoubtedly contributed to the failure of ARDS clinical trials and have limited the success of finding predictive and prognostic biomarkers that have gained broad clinical adoption. These shortcomings have created an opportunity for the application of metabolomics to ARDS. However, the success of metabolomics in ARDS will hinge on its ability to differentiate patient phenotypes within the ARDS diagnosis and to identify patients at risk for developing ARDS, neither of which, to date, have been accomplished. Despite the potential informative nature of the metabolome, few experimental and clinical studies of ARDS metabolomics have been conducted and, to date, most of them are feasibility studies, the goal of which has been to differentiate lung injury from health.

**Table 1:** **The Berlin definition of acute respiratory distress syndrome**.

Timing	Within 1 week of a known clinical insult or new or worsening respiratory symptoms
Chest imaging^a^	Bilateral opacities – not fully explained by effusions, lobar/lung collapse, or nodules
Origin of edema	Respiratory failure not fully explained by cardiac failure or fluid overload
	Need objective assessment (e.g., echocardiography) to exclude hydrostatic edema if no risk factor present
**Oxygenation^b^**	
Mild	200 mm Hg <PaO_2_/FIO_2_ <300 mm Hg with PEEP or CPAP ≥5 cm H_2_O^c^
Moderate	100 mm Hg <PaO_2_/FIO_2_ <200 mm Hg with PEEP ≥5 cm H_2_O
Severe	PaO_2_/FIO_2_ <100 mm Hg with PEEP ≥5 cm H_2_O

*CPAP, continuous positive airway pressure; FIO_2_, fraction of inspired oxygen; PaO_2_, partial pressure of arterial oxygen; PEEP, positive end-expiratory pressure*.

*^a^Chest radiograph or computed tomography scan*.

*^b^If altitude is higher than 1000 m, the correction factor should be calculated as follows: [PaO_2_/FIO_2_% (barometric pressure/760)]*.

*^c^This may be delivered non-invasively in the mild acute respiratory distress syndrome group*.

#### Metabolomics Studies in Experimental Models of ARDS

A challenge in ARDS research is the absence of a translational experimental model of the disease (96, 106, 107). Rodent models do not accurately mimic the human disease and promising pre-clinical data so far have not lead to success in clinical trials. Despite this limitation a number of metabolomics studies have been conducted in rodent models of ARDS. Overall, the findings from experimental ARDS metabolomics studies have not informed of novel processes and appear to be disparate because numerous different model systems, sample types, and analytical platforms have been utilized, each with differing metabolic changes (Table 2).

**Table 2:** **Summary of metabolomics studies conducted in experimental ARDS models**.

Model system	Experimental ARDS	Sample type	Analytical platform	Metabolic changes	Reference
Rat	VILI	Lung tissue	High resolution magic angle spinning 1D ^1^H-NMR	↑ Lactate	(119)
				^↓^ Glucose	
				^↓^ Glycine	
		BAL	1D ^1^H-NMR	↑ Glucose	
				↑ Lactate	
				↑ Acetate	
				↑ 3-OHB	
				↑ Creatine	
		Serum	1D ^1^H-NMR and HPLC-MS	↑ Ptdcholine	
				↑ Oleamide	
				↑ Sphinganine	
				↑ Oxo-hexadecanal	
				^↓^ Lyso-ptdcholine	
				^↓^ Sphingosine	
Rat	IT or IV LPS	Exhaled breath	GC-MS	^↓^ Hexanal	(121)
			E-nose	^↓^ Pentadecane	
				^↓^ 6, 10-dimethyl-5, 9-undecadien-2-one	
Rat	VILI	Serum	CE-MS	↑ Choline	(57)
				↑ Ornithine	
				↑ ADMA	
				^↓^ Isoleucine/leucine	
				^↓^ Arginine	
Mouse	IL-1β + TNF-α	Lung tissue	1D ^1^H-NMR	^↓^ ATP	(138)
				^↓^ Energy charge	
				↑ Lactate:glucose ratio	

*VILI, ventilator-induced lung injury; BAL, bronchoalveolar lung lavage; IT, intratracheal; IL-1β, interleukin-1β; TNF-α, tumor necrosis factor-α; 1D ^1^H-NMR, one dimensional proton nuclear magnetic resonance; HPLC-MS, high performance liquid chromatography-mass spectroscopy; GC-MS, gas chromatography-mass spectroscopy; CE-MS, capillary-electrophoresis-mass spectroscopy; OHB, hydroxybutyrate; ptd, phosphatidyl; ADMA, asymmetric dimethyl arginine*.

In a study that utilized male Sprague-Dawley rats, Izquierdo-Garcia et al. used a VILI model of ARDS (108). This involves a repetitive cyclic stretch and over-inflation of the lungs, which leads to diffuse cellular infiltration, inflammation, loss of membrane permeability, activation of the coagulation system, and cell death (109) that is indicative of the exudative phase of ARDS (Figure 11). The found metabolic changes induced by VILI in the lung tissue, BAL, and serum are shown in Table 2. Importantly, the metabolites in the BAL and lung tissue were associated with markers of the ARDS phenotype including peak inspiratory pressure, PaO_2_, and a histologically derived lung injury score. However, there was no association between these indices and the relative intensity of detected serum metabolites. Collectively, the results of this preliminary, qualitative metabolomics study implicated a shift in cell energy metabolism as evidenced by ARDS-induced changes in glucose and lactate in lung tissue and the BAL and possible disruption of cell membrane integrity based on the changes in serum metabolites as well as decreased glycine in the lung tissue. The magnitude of these metabolic changes were related to lung injury severity suggesting that the pathways associated with these metabolites may provide insight in the pathogenic mechanisms that underlie ARDS.

In a lipopolysaccharide (LPS)-induced ARDS model in male rats, Bos et al. utilized a novel collection and pattern recognition tool (eNose, Comon Invent, Delft, Netherlands) in parallel with GC-MS to capture and measure metabolites in exhaled breath (110). This technique permits the assessment of volatile metabolites that are present in exhaled breath (43, 111), and the application of which could be the prediction or early diagnosis of ARDS because it may be a more sensitive test than currently used diagnostic parameters (e.g., chest radiograph, PaO_2_/FIO_2_ ratio). It may also be useful to longitudinally track drug treatment response. The eNose is a pattern recognition tool that works by reversibly binding a broad range of volatile organic compounds to seven metal-oxide sensors which results in a change in electrical resistance.

For this work, IV (as a model of indirect lung injury) or intratracheal (IT; as a model of direct lung injury) LPS (96, 105, 106) was administered to anesthetized rats that received either low (0) or high (5 mm Hg) PEEP. In exhaled breath condensate, IV and IT LPS induced changes in 21 and 14 GC-MS detected metabolites, respectively. The eNose was effective in discriminating LPS treated and control animals. The found differences between LPS-treatment and controls pointed to alterations in metabolites (Table 2) associated with oxidative stress, which is consistent with the known etiology of ARDS. While the overall metabolomics findings from this study are limited to LPS exposure in an experimental model, this report was the first to demonstrate the utility of exhaled breath as a viable, non-invasive biofluid for early detection of ARDS-induced changes in lung metabolomics. In addition, the eNose strategy successfully detected lung injury early in the course of illness, although not as early as GC-MS, following IV administration of LPS. In the clinic, early identification of patients at risk for ARDS could have a significant impact on improving morbidity and mortality.

In a method development and validation study, Naz et al. showed in a rat model of VILI that a CE-MS metabolomics assay successfully identified 18 compounds associated with lung injury in serum (53). In this study, five metabolites of ARDS were identified (Table 2). Of these, the decline in arginine associated with ARDS has previously been reported, and its supplementation has been shown to reduce inflammation (112). Arginine is converted to urea and ornithine by arginase, the latter of which is a precursor of proline, the primary amino acid in collagen. A reduction in arginine and the associated increase in ornithine suggest that arginase activity is increased in this model of ARDS and contributes to the enhanced collagen deposition and cell proliferation that is known to occur during the proliferative and fibrotic phases of ARDS (Figure 11). Interestingly, elevated levels of ADMA, an arginine analog and inhibitor of nitric oxide synthase (113), can uncouple NOS perpetuating the production of superoxide anion (${\text{O}}_{2}^{-}$) (114, 115). In turn, because ADMA is a competitive inhibitor of NOS, reduction of nitric oxide in the presence of ${\text{O}}_{2}^{-}$ can lead to the production of peroxynitrite (116). In this model of ARDS, which is indicative of VILI, the metabolic consequences of increased ADMA may contribute to lung inflammation but no measurements or phenotyping of lung injury were done. This study also introduced the possibility that the found increase in choline may represent a protective mechanism in this VILI model of ARDS. In addition, the “fingerprinting” approach used in this study serves as a metabolomics strategy that could be tested as a screening tool for patients in the ICU at risk for the development of ARDS.

In one of the first studies of experimental ARDS lung metabolomics, we utilized a cytokine-induced lung injury model to test the extent of the temporal association between the visual phenotype of inflammation in the lungs [as measured by magnetic resonance imaging (MRI)] and changes in the lung metabolome (117). We found that cytokine-induced lung inflammation resulted in a decreased energy state as evidenced by ATP depletion, energy balance, and energy charge levels (Table 2). In addition, there was a significant increase in glycolytic activity (elevated lactate-to-glucose ratios). This metabolic pattern normalized 24 h after the induction of injury. The spectrum of ALI spans from mild interstitial edema (reversible damage) to extensive cellular injury (irreversible damage) (118) (Figure 11). Presently, biomarkers that differentiate the two extremes have not been identified but if found, could represent a powerful experimental and clinical tool to distinguish the range and extent of pulmonary injury. The value of this study was that it demonstrated the association between phenotypic and metabolic changes which is an important first step in biomarker discovery (59). In doing so, MRI and metabolic NMR spectroscopy may enhance the development of more robust and predictive longitudinal models of experimental lung injury.

In summary, there is a common, overarching theme from these studies: lung injury results in a perturbation of energy and oxidative stress metabolism, the magnitude of which may reflect the severity of the damage. This is evidenced by changes in a broad range of metabolites associated with these processes, which are consistent with what is presently known about human ARDS. However, in aggregate, these studies have not lead to advancements in the experimental modeling of ARDS that is needed to enhance translation to the clinical situation and they have not informed of previously unrecognized metabolic pathways that may be relevant to ARDS pathogenesis or severity.

#### Clinical Metabolomics Studies in ARDS

Very few clinical metabolomics studies have been conducted in patients with ARDS, and no studies have tested the predictiveness of a metabolomics strategy in patients at risk for developing ARDS. There are, however, a number of studies that have demonstrated the feasibility and utility of metabolomics as an approach for biomarker discovery in ARDS. Like studies in the experimental arena, the future of a metabolomics approach to clinical ARDS will rely on its ability to tell clinicians something they do not already know using presently available clinical tools. This includes prediction of onset as well as differentiation of ARDS phenotypes.

In a study of mechanically ventilated patients, Schubert et al. demonstrated the utility of exhaled breath as a sample for metabolomics analysis (119). In mechanically ventilated patients with and without ARDS, volatile compounds captured on a charcoal filter introduced to the ventilation system were assayed by GC-MS. The ARDS (as defined by AECC) patient group had a range of underlying etiologies that included pancreatitis, sepsis, pneumonia, and trauma. These patients produced over 50% less isoprene than patients without ARDS (21.8 versus 9.8 nmol/m^2^/min) although the variance across both groups of patients was high such that the 95% CI of the medians overlapped. Isoprene is the most abundant hydrocarbon in human breath, and it is primarily generated via the mevalonate pathway of cholesterol biosynthesis (120). The concentration of isoprene in the breath is known to be highly variable, and in the context of ARDS, isoprene levels may decline due to a reduction in cholesterol levels that may be associated with disease severity (121, 122).

The utility of exhaled breath as a viable sample for ARDS metabolomics has been furthered by Bos et al. (111). They found that three metabolites, octane, acetaldehyde, and 3-­methylheptane, discriminated ARDS from non-ARDS patients. The diagnostic accuracy was increased by the addition of the Lung Injury Prediction Score (LIPS) (123) but not by the Acute Physiology and Chronic Health Evaluation (APACHE) II (124) or the Simplified Acute Physiology Scores (SAPS) II (125), all of which are measures of disease severity. Notably, this study did not find any difference in isoprene levels between ARDS and non-ARDS patients. This may due to methodological differences between the two studies and the known variability in the measurement. In addition, exhaled breath isoprene levels can be influenced by other factors such as mechanical ventilation, use of anesthesia and gender (126). ARDS was, however, associated with higher concentrations of breath octane, which was more strongly related to the diagnostic model than any other of the detected volatile metabolite. Octane is a known end product of lipid peroxidation, one of the degenerative processes caused by oxidative stress (127, 128). In addition to octane, the authors reported that acetaladehyde and 3-methylheptane were predictive of ARDS. This is an ambitious conclusion given the sample size of this study and given that there are a number of sources of acetaldehyde including bacteria that may not be specific to ARDS (129, 130) and there is no apparent source of 3-methylheptane in humans (131). Nevertheless, the exhaled breath metabolomics signature is one that reflects oxidative stress.

Assessment of the local lung environment may provide more detailed metabolic information than what is reflected in the blood. However, metabolomics of the lung environment is challenging because it is unclear which type of sample is optimal, and samples are difficult to obtain. Exhaled breath presently requires introduction of specialized equipment into the ventilation scheme and the acquisition of BAL requires the invasive procedure of bronchoscopy. Until recently, the utility of the BAL as a biofluid for ARDS metabolomics was not known. In general, it is a manufactured sample generated by the instillation of normal saline into the airways, which results in a sample with high protein and salt content and low metabolite levels, which limits the utility of 1H-NMR. We demonstrated the utility of BAL as a metabolomics biofluid by assaying samples from patients with ARDS and healthy controls using a newly developed untargeted LC-MS metabolomics assay (51). Using RPLC and HILIC-MS, we identified 26 and 18 endogenous metabolites, respectively, that differentiated ARDS from health. These included lactate and other metabolites associated with energy metabolism such as citrate, creatine, and creatinine, which we previously showed to be increased in the plasma of patients with ARDS (2). These findings demonstrated the utility of BAL as a biofluid for LC-MS metabolomics, and while the objective of the work did not include introducing ARDS biomarker candidates, we did make informative observations about the lung metabolome during ARDS. These included a found decline in phosphatidylcholine, the primary phospholipid of pulmonary surfactant, which has been shown to be inversely related to inflammatory-cell mediated lung injury (132). However, the strongest found metabolic signal was from guanosine metabolism. This was evidenced by a 41-fold increase in hypoxanthine, a 19-fold increase in xanthine, and a 4-fold increase in guanosine. We did not detect uric acid, but increases in all its precursor molecules provide evidence that the pathway was activated. Uric acid has previously been shown to be a major “danger signal” in the lung contributing to cell-death-induced acute inflammation, and its production is via xanthine oxidase, which is a known ${\text{O}}_{2}^{-}$-producing enzyme.

Taken together, the metabolomics data generated to date from both experimental and clinical studies of ARDS implicates perturbations in energy and oxidative stress metabolism, which is consistent with what is already known about ARDS. Very few clinical studies with ample samples sizes have been conducted. Importantly, multi-center, prospective studies with robust validation testing have not yet been done. To date, the body of knowledge of ARDS metabolomics has been generated from small studies that have demonstrated feasibility and provide promise that the field has potential for discriminating the ARDS phenotype as well as distinguishing lung injury severity. As the field moves forward, progress in metabolically detailing ARDS heterogeneity will be needed in order to bring an “added value” in the phenotyping of ARDS and for providing needed aid in designing clinical trials aimed at testing prevention and treatment strategies in ARDS patients. This is particularly relevant since the National Lung, Heart and Blood Institute recently launched an effort aimed at the prevention of ARDS called the Prevention and early Treatment of Acute Lung Injury (PETAL) network.^5^

### Monitoring Exposure of Lungs to Environmental Insults

Poor air quality in environmental and occupational settings has detrimental effects on the respiratory health of adults and children. According to the World Health Organization, seven million deaths were attributed to the combined effects of ambient and household air pollution in 2012 alone (133). Among these, 8% were due to acute lower respiratory disease, 17% to chronic obstructive pulmonary disease (COPD), and 6% to lung cancer. The remaining deaths were attributed to ischemic heart disease (36%) and stroke (33%). Many of these were premature deaths were due to the burning of solid fuel for heating and cooking, mainly in developing countries. In 2013, the International Agency for Research on Cancer (IARC) established that air pollution was carcinogenic to humans. Specifically, increased exposure to particulate matter was related to an elevated risk of lung cancer (134). In 1998, the National Institute for Occupational Safety and Health produced a report on respiratory diseases in the United States from 1982 to 1993 due to occupational exposure (135). The leading respiratory diseases resulting in mortality were COPD, pneumonia, and lung cancer, with more than 500,000 annual deaths for these diseases combined in the US (135).

Evaluating the effects of environmental insults on respiratory diseases requires proper monitoring of environmental and occupational exposures to environmental contaminants. This is normally performed by collecting air samples in breathing zones of individuals at risk. However, this technique may not always be convenient, and does not accurately reflect the quantity of airborne samples that are inhaled or consumed by exposed individuals. Thus, biomonitoring has become increasingly popular, in which samples are obtained from exposed individuals to determine the impact of airborne particles on health. Recent research has focused on biomarkers related to oxidative stress in the blood of subjects exposed to ambient particulate matter, where a positive association was found between indoor and outdoor concentrations of polyaromatic hydrocarbons (PAHs) and blood levels of sTNF-RII and IL-6 (136).

Welding fumes have also been known to have adverse effects on health, and recent studies have looked at biomarkers of exposure for welders that correlate with increased inflammatory markers (138, 137–140). Blanc et al. (137) have shown an increase in the pro-inflammatory cytokines TNF, IL-6, and IL-8, and polymorphonuclear leukocytes in bronchoalveolar lavage with increasing time of exposure to welding fumes in healthy subjects. In another study, a positive correlation between iron and leukotriene B_4_ was found in non-smoking welders, while a similar correlation was observed between iron, prostaglandin E2, and 8-isoprostane in smoking welders (138). Urine levels of 8-hydroxydeoxyguanosine (8-OHdG) were also increased in boilermakers from pre- to post-shift (139).

Metabolomics is a novel approach that has been proposed to monitor exposed individuals and to develop biomarkers of exposure. Recent research has focused mainly on exposure to heavy metals (141–146), dioxins (147–149), vinyl chloride (150, 151), pesticides (152, 153), smoking (154–156), and welding fumes (157–159). These studies examined metabolite profiles in serum, plasma, or urine samples from control and exposed individuals, and each was able to determine significant metabolic changes in body samples in response to environmental exposure to toxic substances. Metabolites were measured by NMR, LC-Q-TOF-MS, GC-MS, and UHPLC-QTOF-MS analyses and subjected to multivariate statistical comparisons. In general, the findings from these studies showed that samples from control groups clustered in a different region of PCA or OPLS-DA score plots from that of exposed individuals.

Of particular interest are the studies on exposures to cadmium, cigarette smoke, and welding fumes, as these contaminants are known to cause adverse respiratory health effects (160–164). In the case of environmental exposure to cadmium, one study using NMR spectroscopy in urine samples observed an increase of citrate concentrations in exposed subjects (141) while another study using LC-Q-TOF-MS and GC-MS showed elevated concentrations of l-glutamate, l-cysteine, l-tyrosine, *N*-methyl-l-histidine, l-histidinol, taurine, phenyl-acetyl-glutamine, hippurate, α-pyroglutamic acid, d-galactose, myo-inositol, xanthine, urea, deoxyadenosine monophosphate, creatine, creatinine, 7-α-hydroxyprogesterone, tetrahydrocortisone, estrone, and corticosterone in subjects presenting with high urinary cadmium (142). A similar study showed elevated concentrations of myo-inositol and a decrease in citrate for subjects presenting symptoms of cadmium toxicosis (143). Occupational exposure to lead, cadmium, and arsenic demonstrated an increase in 1-­methylhistidine, phenylalanine, low-density lipoproteins, tyrosine, and unsaturated fatty acids, and a decrease in very low-density lipoproteins and glutamate in the serum of exposed subjects using NMR spectroscopy (146).

Two studies were performed using MS to evaluate metabolites in the serum of smokers (156, 157). The first one found an increase of 23 lipid metabolites (156), and the second one observed a change in both lipid and amino acid metabolism in both genders (157). Interestingly, smoking cessation seemed to reverse some of these metabolites to baseline (157).

Only two studies have been performed examining occupational exposure to welding fumes (158, 159). The first study was performed on urine samples of welders using NMR spectroscopy and observed an increase in glycine, taurine, TMAO/betaine, serine, *S*-sulfocysteine, hippurate, gluconate, creatinine, and acetone, and a decrease in creatine (158). The second study was performed on plasma samples from boilermakers, which were analyzed using MS, and a decrease in eicosapentaenoic acid and docosapentenoic acid was observed in these participants (159).

Therefore, these reports suggest that metabolomic measurements may be useful for the generation of appropriate biomarker candidates that allow monitoring of exposure levels of susceptible individuals. However, these studies show variability in metabolite profiling depending upon the technique used or the media analyzed. Based on the few environmental studies conducted using metabolomic techniques, we are some way from validating these approaches as each technique requires careful calibration and appropriate use of quality assurance/quality control samples to ensure that measurements are robust and reproducible. Provided that appropriate quality assurance/quality control is carried out in each study, it may be possible to elucidate patterns of metabolite changes that can be used as biomarkers of environmental exposure to toxins.

## Conclusion

In summary, we have reviewed the rapidly expanding field of metabolomics and its application to acute lung diseases. Metabolomics is an important component of systems biology that has enormous clinical potential in the development of biomarkers and as a novel approach to understanding disease mechanisms. Metabolomics allows us to generate a snapshot of all the metabolites present in a biological sample, and to follow rapidly changing trends in metabolites over time in a way that cannot be captured by genomics or proteomics. These changes may be monitored by the application of NMR or MS-based approaches. The challenge for the application of metabolomics to acute lung diseases rests with whether it will be able to identify more precise patient phenotypes that are not presently recognized by currently available clinical tools. The extent of the predictive and prognostic value of a given set of metabolites (e.g., biomarker credentials) will be required for optimal patient selection for clinical trials and ultimately for clinical decision making (14, 15) that will be needed to realize precision medicine. To date, urine metabolomics shows promise for rapidly differentiating pneumonia pathogens that is needed for timely antibiotic selection. However, for ARDS, metabolomics data that enable the distinction of susceptible patients and ARDS severity, are lacking. Analytically, there is a need to improve the sensitivity of NMR analysis and its reproducibility across centers. For MS-based approaches, new developing strategies to address the large number of unknown metabolites are being tested. With these challenges in place, we look forward to a future of increasingly sophisticated analyses of biological samples that will enhance our capability for diagnosing and monitoring human lung diseases.

## Author Contributions

All authors listed, have made substantial, direct, and intellectual contribution to the work, and approved it for publication.

## Conflict of Interest Statement

The authors declare that the research was conducted in the absence of any commercial or financial relationships that could be construed as a potential conflict of interest.

## Footnotes

^1^www.chenomx.com

^2^http://metscape.ncibi.org/

^3^http://www.cytoscape.org/

^4^http://metabolomicssociety.org/resources/metabolomics-software

^5^http://petalnet.org/
